# Implementation of Drive-Through Testing for COVID-19 With Community Paramedics

**DOI:** 10.1017/dmp.2021.46

**Published:** 2021-04-16

**Authors:** S. Tyler Constantine, David Callaway, Jeremy N. Driscoll, Stephanie Murphy

**Affiliations:** Atrium Health’s Carolinas Medical Center, Charlotte, NC, USA

**Keywords:** community paramedicine, COVID-19, drive-through testing, mobile integrated health, pandemic

## Abstract

**Objective:**

In this manuscript, we discuss the implementation and deployment of mobile integrated health and community paramedicine (MIH/CP) testing sites to provide screening, testing, and community outreach during the first months of the 2019 coronavirus disease (COVID-19) pandemic in the metropolitan region of Charlotte, North Carolina. This program addresses the need for an agile testing strategy during the current pandemic. We disclose the number of patients evaluated as “persons under investigation” and the proportion with positive severe acute respiratory syndrome coronavirus 2 (SARS CoV-2) results from these sites. We describe how the programs were applied to patient care and include considerations on how additional staffing, scalability, and flexibility of these services may be applied to future patient and health care crises.

**Methods:**

This is a descriptive report of the implementation of MIH/CP test sites in our health care system’s early response to the COVID-19 pandemic in March 2020. Retrospective data on the number of patients and their associated demographics are reported here as raw data. No statistical analysis was performed.

**Results:**

Between March 15, 2020, and April 15, 2020, our 6 MIH/CP test sites evaluated 4342 patients. Of these, 401 patients (9.2%) had positive test results, 62.8% of whom were women. The estimated duration of each patient encounter under investigation was 3 to 5 minutes. The paramedics were able to perform a brief history, specific physical examination, and screening for signs of hypoxemic respiratory failure. There were no cases of accidental exposure or failure of personal protective equipment for the MIH/CP paramedics.

**Conclusions:**

In our health care system, we pivoted the traditional MIH/CP model to rapidly initiate remote drive-through testing for COVID-19 in pre-screened individuals. This model allowed us to test patients with suspected COVID-19 patients away from traditional health care sites and mitigate exposure to health care workers and other patients.

## Introduction

Determining effective strategies for testing patients with suspected 2019 novel coronavirus disease (COVID-19) remains a challenge as the outbreak continues to spread in the United States. The organism is thought to be highly contagious, with a reproductive number (R_0_) reported at 2.28 from the Diamond Princess cruise ship, and variably reported to be between 1.4 and 6.49.^1,2^ Person-to-person transmission likely takes place when infected persons are in close proximity to others. It is therefore imperative that potentially infected persons exhibiting symptoms of COVID-19, who do not otherwise require acute medical intervention and/or hospitalization, remain in isolation and avoid hospital emergency departments (EDs) and other health care settings. According to the Centers for Disease Control and Prevention (CDC), screening for upper respiratory illness should ideally take place before patients enter a health care facility. The CDC also supports the use of telemedicine to minimize disease transmission and exposure to health care workers.^3^

Health systems worldwide have developed strategies to perform testing of suspected COVID-19 patients outside of clinical settings to augment containment strategies and reduce the likelihood of disease transmission within the health care system. In London, 1 such strategy involves dispatching health care personnel to suspected patients’ homes for in-home testing after an initial telemedicine screening to determine appropriateness for screening.^4^

The concept of drive-through medicine has been previously reported. In 2010, Weiss et al.^5^ modeled a drive-through testing model to evaluate efficacy in the case of a pandemic (in that case, influenza-H1N1). They found that ED physicians could rapidly evaluate and determine appropriate management of patients with influenza-like-illness in a drive-through setting.^5^

Drive-through testing has been successfully deployed in South Korea and the United States.^6^ In Colorado, an initial deployment that was reported in the lay media was quickly inundated with persons wishing to be tested.^7^ Building on the H1N1 models and rapid cycle review of global COVID-19 responses, our team recognized the need for more robust operational capabilities to support large scale deployment of drive-through testing.

Mobile integrated health (MIH) and community paramedicine (CP) programs perform a wide array of non-emergent paramedic functions, such as post-discharge care, chronic disease management, and assuring proper health care resource utilization.^8^ Our hospital system leadership recognized that robust MIH/CP services could provide remote COVID-19 testing that reduced patient and staff exposure at brick-and-mortar clinical facilities, increased community access to testing by strategic placement of the drive-through sites, and supported broader COVID-19 mitigation strategies such as virtual care delivery.

We are not aware of previous efforts to use MIH/CP services to implement drive-through testing in pandemic scenarios. We report on our experience developing this testing strategy, as well as recommendations for communities that may wish to deploy a similar testing strategy.

## Methods

### Program Description

Our MIH/CP program serves patients living in North Carolina and South Carolina under a large, multi-hospital health care system. The paramedics operate in urban, suburban, and rural areas with a wide geographic reach. Similar to other MIH/CP programs in the United States, ours focuses on reducing readmission for high-risk discharges, reducing ED visits and admissions by high-health-care users, and assisting with management of acute and chronic disease in various populations referred from the health care system’s subspecialties. Paramedics are licensed by the state, which currently does not offer formal licensure in community paramedicine.

In our health care system, these paramedics play a crucial role in bridging the gap between out-of-hospital care and brick-and-mortar care, especially for patients with chronic health conditions and comorbidities. They have a wide geographic reach, a broad and adaptable skill set and close medical oversight from medical directors with training in emergency medicine and internal medicine, along with the ability to link patients in the field with physicians using existing telemedicine platforms. These attributes made the MIH/CP service a logical choice to perform clinical assessment and COVID-19 testing at the drive-through sites.

The program almost exclusively sees patients from the health care system and does not work directly with the local 911 emergency medical services nor have any direct relationship with local city or county public health services. At present, the majority of the program’s patients are referred from a transition clinic, which focuses on reducing hospital readmission for patients recently discharged from acute care facilities. In 2019, the MIH/CP program completed 2182 patient encounters.

### Patient Selection

Our MIH/CP program has worked closely with the health care system’s infectious disease (ID) and infection prevention (IP) teams in pre-screening patients for testing. Screening occurred via a mixture of telephonic and in-person evaluation. We initially observed that most screenings occur via telephone, although data are not currently available to confirm this. Screening criteria that determined the need for testing evolved as disease epidemiology changed. An example of early screening criteria (April 29, 2020) is demonstrated in Box 1. Patients were eligible for screening without regard to age, sex, race, or ethnicity, and occurred with the approval of an ID specialist when necessary. Anyone with concerns about being exposed to, or having the symptoms of, COVID-19 could be screened for testing. The initial patient population for this process consisted mostly of employees of the health care system with concerning travel or exposure history who were referred by occupational health. As capacity increased and processes matured, the team added additional patient populations, including primary care and other ambulatory care referrals. The system also developed an online option for patients to be screened digitally, with subsequent referral for testing as needed based on the screening results.

Box 1. PUI screening criteria.

The criteria for approved PUI testing for Wednesday April 29

- Influenza-like Illness (ILI) = fever 100.4 F or greater or subjective fever, cough or shortness of breath in any patient in any care setting
- Any admission from long-term care (LTC), homeless shelter/encampment or jail/prison
- Adult patients who are unable to give history and relay symptoms of COVID-19
- Hospitalized patients with acute stroke of unknown cause or radiographic evidence of large vessel occlusion stroke requiring intervention

New Additions that DO NOT require approval:

- Inpatients discharged to congregate living facility (order test within 48 hours of DC)
- Preoperative/preprocedural testing as approved by service line guidance
- Consider testing high-risk individuals with any of the CDC expanded symptoms:
  ○ Chills/repeated shaking
  ○ Muscle pain
  ○ Headache
  ○ Sore throat
  ○ New loss of taste or smell
  High-risk individuals:
  ○ Health care workers/first responders
  ○ Individuals who live or work in congregate living settings
  ○ Immunocompromised
  ○ Age > 65 years
  ○ Those living with high-risk individuals
  ○ Pregnancy
- Patients with mild symptoms and low health risk do not necessarily need testing based on clinical judgment but should have the order “suspected COVID”

### Initial Considerations

In keeping with typical disaster management strategies in the United States, our health care system deployed an emergency operations center (EOC) and formalized an incident command (IC) structure early in the pandemic. Initial conversations between MIH/CP Medical Direction, Medical-Technical Specialist – Emergency Medicine, and Mobile Medicine leaders highlighted a need for remote testing away from acute care facilities to best identify and isolate suspected COVID-19 patients and protect health care workers and non-COVID-19 patients in the hospitals. We determined that the MIH/CP team could redeploy some of its personnel, equipment, and logistical resources to quickly set up mobile testing sites for suspected COVID-19 patients. The Medical Branch Director tasked MIH/CP leadership with establishing a pilot within 24 hours. The first test site was established on March 11, 2020, screening and testing 12 patients.

### Implementation

The MIH/CP test site program was initiated in early March 2020 prior to the identification of any known COVID-19 cases in our state. The IC conveyed an appropriate sense of urgency to quickly start a pilot program and begin testing persons under investigation (PUIs). The initial MIH/CP pilot was intended to create 1 testing site, with an aim to scale to additional testing sites as needed. Initial testing only involved symptomatic employees of the health care system. The IC estimated that 2 to 3 testing sites would ultimately be required.

### Staffing

Leadership in the IC structure initially planned to use existing MIH/CP staffing resources on an ad hoc basis, with other clinical and non-clinical staff added as needed for higher volumes. Initial staffing included 2 MIH/CP paramedics conducting PUI testing, the MIH/CP manager in a non-clinical administrative oversight role, and another MIH/CP paramedic functioning as a supervisor. As discussed in the next section on scalability, this staffing evolved rapidly to accommodate additional sites and increases in volume. Staffing roles are outlined in Appendix A, an active document used to help track current staff responsibilities. This document evolved rapidly to reflect changing staffing needs and test-site design.

### Scalability

The initial deployment underwent continuous rapid-cycle quality improvement. Early test-kit and viral media shortages temporarily threatened test-site scalability; however, our system was able to secure the necessary supplies within the first 2-week operational period. Additional clinical and non-clinical staff were involved in the project to adapt to increasing testing volumes. Over the first 5 weeks of the local pandemic response, the MIH/CP test-site operations expanded from 1 site to 6 full-time sites, operating 7 days per week for about 6 hours per day. This expansion supported a system-wide strategy to leverage mobile testing sites, telehealth, and virtual care to create a novel surge strategy and expanded acute care capacity. Given our health care system’s massive geographic catchment area, the expansion accommodated the complex network of hospitals and markets operating across multiple counties in North and South Carolinas.

The cross-functional MIH/CP team established and evolved standard operating procedures at the test sites that allowed for rapid scaling of new test sites at the discretion of the EOC. The close communication between field and strategic leadership allowed for near real-time adaptation of processes as new lessons were learned at geographically distinct sites. Barriers to scaling the successful MIH/CP drive-through models include staff availability, equipment, and access to the various materials required for test acquisition and safe transport. Staffing and equipment availability have been the primary barriers to adding new test sites.

### Geography

The pilot focused on the local metropolitan area because of pre-existing resources, greater population density, and anticipated higher need for testing in that locale. The initial testing site was chosen because it had a large available parking lot that was already in use by the health care system. This location was intentionally positioned distant from any health care facility to avoid congestion at the brick-and-mortar facilities and decrease disease exposure to other patients and health care personnel. This site was easily accessible from local highways and had minimal foot and automobile traffic. Later, a warehouse at this initial site, also already in use by the health care system, was used to create an IC center, which coordinated operations and logistics at all test sites.

A second location was established on Day 4 of the pilot in a neighboring county on the opposite side of the metropolitan area to expand access for patients. This site was located at a racetrack near, but not adjacent to, a local acute care facility. The racetrack was identified as an optimal location for a testing site due to large, unused infrastructure for staff and patient access. The subsequent 4 MIH/CP test sites deployed were tactically located by the EOC to help support anticipated testing needs. Ultimately, a total of 6 test sites were established, modeled off the initial sites. Locations were chosen to maximize geographical spread and minimize patient driving distance. The health system EOC chose locations in order to maximize patient access to testing, accounting for drive time, access to public transportation, proximity to referral health centers, and community social vulnerability indices.

After the initial MIH/CP drive-through sites were established, the EOC quickly identified racial and ethnic disparities in COVID-19 positivity rates, which were reported in the lay press.^9^ The system adapted the drive-through testing sites to create a roving test-site model specifically focused on reaching underserved communities for screening and testing. This model is not discussed at length in this report but highlights the ability of our model to pivot toward assisting neighborhoods that are more diverse and have few health care facilities by providing non-referral-based walk-up screening and testing. Patients in this data set do not include the roving test sites, as the time frame for data collection is prior to that model’s initiation.

### Patient Referral

Patients in the MIH/CP testing program were referred via the infectious disease/infection prevention (ID/IP) chain as described in the section on patient selection. After screening, assessment, and determination to be a PUI (recent criteria in Box 1), patients were then referred to the MIH/CP administration via secure e-mail and given instructions on when and where to present for testing. PUIs were also instructed to arrive by vehicle, follow instructions on site, and not to exit the vehicle.

As this process has evolved, patients were eventually screened via a system-wide COVID-19 telephone hotline, facilitated by a public-facing Internet website. This hotline was established to help patients have access to testing and care, which was especially helpful for those patients who did not have primary care physicians (PCPs) and might otherwise present to the ED for testing. Patients could also be pre-screened via their PCP or other ambulatory care sites. Patients were scheduled directly for testing at an MIH/CP test site using digital scheduling software. When testing was indicated after screening, the test was scheduled to be obtained within 24 hours. The patient was registered and the test order was placed at the time of pre-screening via the hotline so a brief clinical evaluation and testing could be conducted rapidly with minimal need for on-site registration, documentation, and ordering.

### Testing Site Infrastructure

In the initial phases of the MIH/CP test sites, an ambulance was parked in a large parking lot, with traffic cones and signage established to direct PUIs to it. Staff established a work area with essential supplies and documentation. This process evolved, and updated MIH/CP test sites were substantially better equipped to manage the flow of patients. Box 2 demonstrates current logistical, supply, and staffing needs to operate one of our MIH/CP test sites.

Box 2. MIH/CP test-site staffing and supply checklist (per site).

*Staffing*

- 1 Registrar (EMR/schedule access)
- 1 Charter (EMR access)
- 1 Clinical (NP Swab Collection: EMT, Paramedic, CNA, RN, RT)
- 1 Clinical (Vitals/Assessment: EMT, Paramedic, CNA, RN, RT)
- 1 Security (depending on site: hospital, contractor, county Sheriff)
- 1 Bus Driver (operates mobile testing unit bus)
- 1 Runner/Cleaner/Site Lead

*Clinical and Non-Clinical Supplies*

- 1 Label printer
- 1 Regular printer
- 3 Laptops (1 with virtual equipment loaded)
- 1 Wireless Internet card
- 2 Tables
- 6 Chairs
- 1 Pop-up tent
- 75 Testing kits
- PPE (1 box of all-size gloves, N95, surgical mask, face shields [4], 1 box of gowns). Each day, this is refilled as needed
- Water
- Hand sanitizer
- Free standing handwashing station or free-standing sanitizer station
- Patient education paperwork
- Employee note
- Lab slips (backup for failed label printer)
- Sani-Wipes
- Cooler with ice (for tests)
- Lab pickup location (twice daily)
- 1 Thermometer
- Thermometer covers (quantity sufficient)
- 1 Pulse oximeter
- 1 Box alcohol prep pads
- Signage (directional and educational)
- Pens
- 3 Clipboards
- 6-12 Traffic cones
- 1 Stat vital sign device (nurse on a stick)
- 3 Trash cans (1 normal trash, 1 shred bin, 1 bio hazard bin)
- Trash bags
- Bio hazard bags for test storage for pickup

### Evaluation and Testing

PUIs arrived by vehicle to the testing site and were directed into a vehicle line with the assistance of on-site security staff. Upon reaching the end of the line, they underwent paramedic evaluation through the window or door of the vehicle while remaining seated. Using a test process protocol (Appendix B), paramedics evaluated the general appearance of the PUI using a triage protocol.

The MIH/CP team developed a protocol that allowed clinicians to provide a more advanced clinical evaluation of adult PUIs at the drive-through sites (Box 3). This protocol employed the objective parameters of the DSCRB-65 screening tool to identify high-risk patients and refer them to a virtual physician evaluation on-site. This protocol also played a role in follow-up care and either virtual or brick-and-mortar hospitalization. The DSCRB-65 is a modified version of the CRB-65 screening tool, which has been previously validated as a predictor of pneumonia severity.^10,11^ It has not been validated for the screening of illness severity for patients with COVID-19 but was used here in an effort to provide an objective indicator of patients who may require more intensive outpatient or inpatient care in virtual or brick-and-mortar hospital.

Box 3. COVID triage protocol (adults).

I. Confirm Patient Registration/Testing Ordered
II. Perform Vital Signs and Calculate DSCRB-65 Score (If patient scores 0-1 and is clinically well appearing, can avoid performing BP at triage site) [Definition of clinically well appearing is patient not having sunken eyes, pallor, dry mucous membranes, appearance of fatigue]
  a. Chronic **D**isease state evaluation by history from patients **1 Point**
    i. Cardiovascular disease – HTN/CHF/CAD
    ii. Respiratory disease – COPD/Asthma
    iii. End Stage Liver Disease, Cirrhosis
    iv. End Stage Renal Disease, on Hemodialysis or Peritoneal Dialysis
    v. Active Cancer
    vi. Uncontrolled diabetes, if has diabetes and average glucoses are greater than 180.
  b. O2 **S**at – Less than 92%, **1 Point**
  c. **C**onfusion – GCS Less than 15 (From baseline for patients with dementia), **1 Point**
  d. **R**R – Count respirations for 30 seconds x2. Greater than 22, **1 Point**
  e. **B**P – Systolic less than 90, **1 Point**
  f. Age greater than 65, **1 Point**
  g. Oral temperature
  h. HR
III. Perform Testing via NP Swab
  a. Label and file as ordered
IV. DSCRB-65 Score of 0-2 Patient proceeds home
  a. If a patient scores a 2 but has any of the following, consult with a provider prior to sending home:
    i. Oxygen saturation less than 91%
    ii. Respiratory rate of greater than 23
    iii. Confusion beyond baseline
V. DSCRB-65 Score of 3 or greater bring patient into tent for further evaluation
  a. Repeat Vital Signs: If at any point patient is in Extremis follow BLS/ACLS algorithms
    i. Temperature
    ii. HR
    iii. RR
    iv. BP
    v. O2 Sat
  b. Virtual Visit with CHG Provider
    i. Disposition pending that evaluation
  c. Perform EPOC testing as ordered by provider
  d. Butterfly exam per provider.
  e. Direct admissions to the hospital will be transported by MEDIC or private vehicle per provider conversation.
  f. Admissions to Virtual Home Hospital receive monitoring kits prior to departure from site. Highlight 24/7 call number.

*Notes*: At the beginning and end of shifts, staff will perform vitals on each other including temperature, heart rate, respiratory rate, blood pressure, and O2 sats. Notify medical directors of any abnormalities for guidance in quarantine and testing.

Pediatric patients were evaluated using pulse oximetry, general appearance, and work of breathing. There was not a specific protocol developed for the evaluation of pediatric patients.

Paramedics were instructed to take notice of any increased work of breathing by the PUI. A reusable fingertip pulse oximeter was deployed for every PUI to evaluate pulse oximetry and heart rate, which were documented in the electronic medical record (EMR).

Paramedics obtained nasopharyngeal (NP) viral swabs per CDC guidelines. Initially, this required separate NP and oropharyngeal (OP) swabs; this was changed to a single swab for both nares as updated by the CDC shortly after test-site creation.^12^ After collection of swabs, patients were given verbal and written instructions on maintaining social distancing and in-home quarantine, pending test results.

MIH/CP paramedics maintained persistent personal protective equipment (PPE) with gown, gloves, and mask with face shield. Gloves were exchanged between every PUI contact following CDC guidelines.^3^ Additionally, the staff maintained hot and cold zones to separate charting, registration, and equipment maintenance activities from patient testing and evaluation.

At the time of publication, COVID-19 polymerase chain reaction (PCR) turnaround time was 12–24 hours. Swabs were batched and picked up twice daily at the testing sites for processing at the hospital system’s core laboratory. Patients received test results from a centralized resource used to ensure notification and appropriate follow-up care.

### Documentation

PUIs were entered into the hospital system’s EMR. Subsequently, a brief note using the MIH/CP’s standard EMR form was entered to document the encounter and vital signs. The CDC PUI and Case Report form^13^ was initially used for each encounter to be submitted to the CDC; however, this was discontinued in favor of system-wide reporting of testing to the CDC via other processes.

Additionally, data on the number of patient encounters at each test site for the preceding 24 hours were reported via nightly e-mail to the leadership team in the form of a situation report.

### Data Analysis

Data from the 6 MIH/CP test sites were evaluated retrospectively between March 15, 2020, and April 15, 2020. Patients were included for this report if they were seen at one of these sites and underwent testing for COVID-19 via an order placed in the EMR. There were no exclusion criteria. Data were tabulated and analyzed to report on patient demographics. Results are reported here without additional statistical analysis.

## Results

Data are not available to indicate the number of patients pre-screened. A total of 4342 patients were tested at our MIH/CP sites between March 15, 2020, and April 15, 2020 (Table 1). The mean age of patients tested was 42.2 years with a standard deviation of 16.8. Ages seen across sites ranged from 3 months to 95 years. Most patients were female (n = 2871; 66.1%) with a similar proportion of females (62.8%) testing positive (see Table 1). Patients who identified as Caucasian made up the largest group tested compared to the next largest group, which was African American (65.6% vs 21.2%). Of all patients, the majority identified their ethnicity as non-Hispanic (85.7%).

**Table 1.** Baseline characteristics of persons under investigation (PUIs) at presentation to MIH/CP test sites

All PUI Patient Characteristics N = 4342		COVID-19 Positive Patient Characteristics N = 401
Age *Mean (SD)*	42.2 (16.8)	44.6 (16.0)
Age Range	3 Months–95 Years	1–86 Years
Gender, Female *N* (%)	2870 (66.1%)	252 (62.8%)
Race Declared Caucasian *N* (%)	2849 (65.6%)	193 (48.1%)
African American	920 (21.2%)	141 (35.2%)
Asian	52 (1.2%)	3 (0.7%)
Native American	29 (0.7%)	4 (1.0%)
Pacific Islander	0	0
Ethnicity Non-Hispanic *N* (%)	3722 (85.7%)	328 (81.8%)
Ethnicity Hispanic *N* (%)	620 (14.3%)	73 (18.2%)

Of patients tested, 401 (9.2%) were found to be COVID-19 positive by NP swab PCR (see Table 1). The average age of patients who tested positive was 44.6 (±16.0), with the majority being female (62.8%). Given most patients identified as non-Hispanic, a similar proportion of those who tested positive (n = 328; 81.8%) were non-Hispanic.

The estimated duration of each PUI encounter was 3–5 minutes. In addition to COVID-19 testing, the paramedics were able to perform a brief history, focused physical examination, and screening for signs of hypoxemic respiratory failure to risk stratify morbidity and mortality from COVID-19 infection.^14^ There were no cases of accidental exposure or PPE failure among the MIH/CP paramedics. Paramedics were able to maintain safe distancing from PUIs apart from obtaining the NP/OP testing swab.

## Discussion

This process of using MIH/CP to provide off-site, drive-through testing for COVID-19 was successful in evaluating 4342 symptomatic PUIs for suspected COVID-19 infection during the specified time period. This was accomplished expeditiously and safely, with no known transmission of COVID-19 to community paramedics performing the testing. The MIH/CP pilot was scaled rapidly to meet expanded community testing requirements. This has allowed for a marked increase in the ability to test large numbers of patients in different geographic areas, limiting the distance that patients need to travel to reach a test site and supporting public health recommendations to maintain maximal physical distancing.

While the authors are aware of other efforts to conduct off-site drive-through medicine for both COVID-19 and other simulated and real-world pandemics, we did not identify published literature describing the re-deployment of community paramedics from existing roles to perform this function. Based on discussions with other colleagues in the field of prehospital medicine, we believe other entities are considering deploying MIH/CP resources for COVID-19 evaluation and testing. MIH/CP serves this purpose well in a pandemic or other disaster situation because it is a ready, prepared mobile asset with a wide range of clinical abilities that can be redeployed on short notice. In our health care system, a rapid shift in the day-to-day functions of the MIH/CP service allowed for an almost immediate implementation of a previously undeveloped drive-through testing strategy.

The percentage of COVID-19-positive patients in our population tested was lower than the CDC’s recent report of 18.4% cumulative positive tests as of April 11, 2020.^15^ The most recent rate of COVID-19-positive patients requiring hospitalization is 0.02%, with the highest rates being seen in patients ages 65 years and older.^15^ We did not specifically evaluate the number of patients tested at our remote test sites who required emergent ED referral or hospitalization. Anecdotally, we know this was an extremely infrequent event, but further research should attempt to quantify this and compare rates of ED use and inpatient hospitalization between the MIH/CP sites and other care and testing sites.

This pilot highlights MIH/CP paramedics as an asset to the COVID-19 pandemic and future pandemics that require patient identification and testing, as well as robust social isolation. Whether this model is reproducible and sensible in other systems is likely an individualized decision depending on the local health care environment. We operate in an urban environment with drive-through testing, and, while this model may be equally viable in rural settings, resource constraints may be applicable. We also suggest that MIH/CP paramedics may be best used to continue serving their usual patient populations, which tend to be of higher medical complexity and at risk for comorbid acute illness as the result of, or irrelevant to, COVID-19 infection during this pandemic time.^16^ In our system, a follow-up component of testing was also provided, though not discussed in detail in this report, that uses our MIH/CP service to conduct home visits in a virtual hospital. Our virtual hospital continues to manage COVID-19 patients, whether suspected or confirmed, without hospitalization at a brick-and-mortar hospital unless clinically required. A multifactorial testing strategy could use MIH/CP paramedics as an important, flexible, scalable asset.

### Limitations and Recommendations

Our pilot evaluated referrals from various sources that were manually pre-screened through ID/IP, which then referred PUIs to MIH/CP. We learned quickly that this process is not scalable when larger numbers of PUIs present for evaluation. A comprehensive telehealth and phone screening system that can be advertised locally, combined with screening protocols and better integration of the EMR, allows for more streamlined testing. This requires health care system resources, massive coordination of logistics, and rapid improvement cycles with quality assurance. For example, we identified early in the process development the importance of digital PUI scheduling to avoid overwhelming the test sites.

We do not have data on patients who were screened but *not* recommended to undergo testing based on current guidelines. These patients were advised to maintain usual isolation precautions and social distancing, and to recontact the screening line or their primary care provider if they have worsening symptoms.

The lay media has widely reported on the lack of availability of PPE, testing kits, and viral media. In its initial stages, we also had a limited supply of viral media and test swabs. However, these limitations were temporary, did not delay starting up the test sites, and we were ultimately able to conduct testing on all subjects with paramedics wearing appropriate PPE at all times. Limited equipment availability may subsequently hamper further efforts if conducted on a larger scale. It is recommended that any efforts using MIH/CP ensure adequate supply of these items, especially with an eye to ensuring paramedic safety performing collection.

## Conclusion

MIH/CP is a valuable resource in providing screening and testing of PUIs with suspected COVID-19 during this pandemic, with potential uses and scale depending on local health care infrastructure and available resources. Proper implementation of such a system to meet the specific needs of the community can prove effective for all aspects of the health care system. Our implementation demonstrates how expanding the health care system’s testing footprint can create more efficient care for patients and improve the safety of the rest of the hospital staff and other non-COVID-19 patients.

## Supplementary material

For supplementary material accompanying this paper visit http://dx.doi.org/10.1017/dmp.2021.46. click here to view supplementary material
