# Supplementary material for: Implementation of Drive-Through Testing for COVID-19 With Community Paramedics
Source: Disaster Med Public Health Prep. 2021 Apr 16:1–7. doi: 10.1017/dmp.2021.46 (PMC8111182; doi:10.1017/dmp.2021.46)
Supplement: Supplementary file 1 [file dmpsup.zip › S193578932100046Xsup001.pdf]

Appendix A. Staffing Roles

COVID-19 Testing Site Job Roles				
	**Site Lead (Paramedic)**	**Clinical Interviewer (PPE Required)**	**Clerical Processor**	**Swabber/ Lab Processor (Clinical - PPE Required)**
**Patient Processing**	Assign Daily Tasks	Collects patient information	Confirm patients on schedule	Swab patient
	Place labels on tubes and worknotes	Collects patient vitals	Check In patient & Print Labels	Provide patient care instructions
	Enter or activate Canopy orders<br>- COVID<br>- RRP	Clean equipment in between patients		Process swab and place in cooler
	Complete Canopy Work-Up (Vitals entry)			
	Stay until last courier pick up has occurred			
	Handle patient triage, as needed			
	Investigate patients arriving unscheduled			
**Other Duties**	Manage Inventory and request supplies as needed	Set up tables and chairs at beginning of day. Clean, sanitize, and put away tables at end of day.		
	Manage escalation process (system issues, etc.)	Clean equipment and supplies beginning and end of shift.		
	Secure equipment and supplies at end of day.	Set up cones at beginning of day. Put away cones at end of day.		
