# Supplementary figures and images for: Implementation of Drive-Through Testing for COVID-19 With Community Paramedics
Source: Disaster Med Public Health Prep. 2021 Apr 16:1–7. doi: 10.1017/dmp.2021.46 (PMC8111182; doi:10.1017/dmp.2021.46)

COVID-19 Testing Site- Patient Testing Process

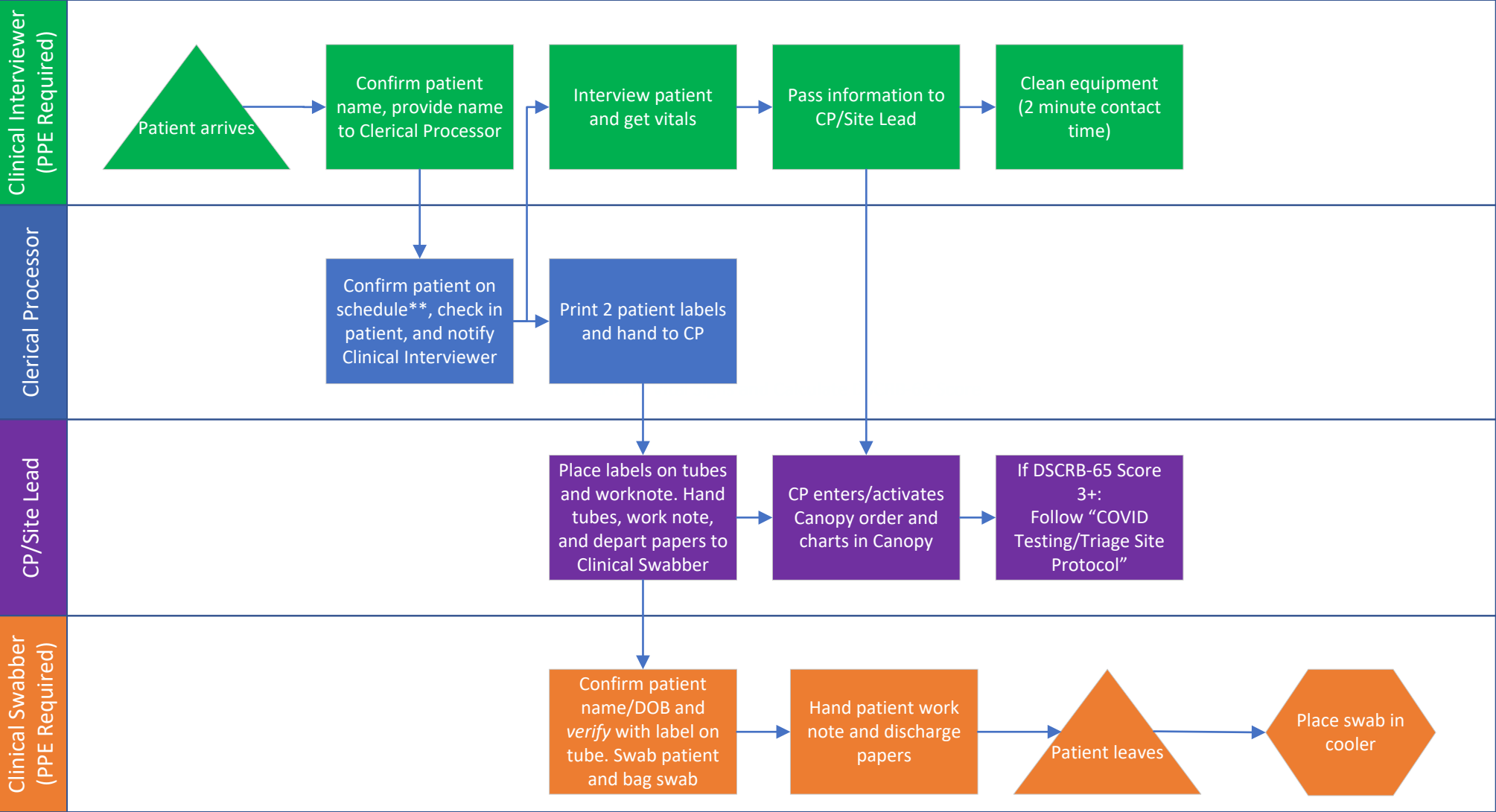

Note:  
"CP" = Community Paramedic

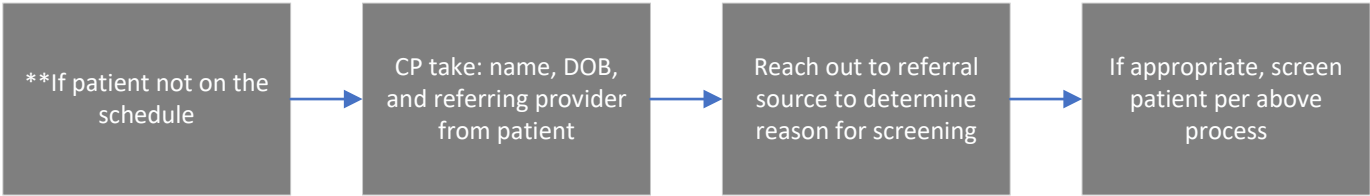

Supplement: Supplementary file 1 [file dmpsup.zip › S193578932100046Xsup002.pdf]
